# Tomorrow Never Comes: The Risks of Procrastination for Adolescent Health

**DOI:** 10.3390/ejihpe14080143

**Published:** 2024-07-26

**Authors:** David Pérez-Jorge, Ana Cristina Hernández-Henríquez, Roshan Melwani-Sadhwani, Anthony Fernando Gallo-Mendoza

**Affiliations:** 1 Department of Didactics and Educational Research, Faculty of Education, University of La Laguna, 38200 La Laguna, Spain; dpjorge@ull.edu.es; 2 Canarian Health Service, Primary Care Service, 38004 Santa Cruz de Tenerife, Spain; alu0100824568@ull.edu.es (A.C.H.-H.); anthall@ucm.edu.es (A.F.G.-M.); 3 Department of Dentistry, Faculty of Health Sciences, University Fernando Pessoa Canarias, 35450 Las Palmas, Spain

**Keywords:** procrastination, adolescents, students, lifestyle, health, habits

## Abstract

This study explores the relationship between procrastination and declining healthy habits among adolescents, a topic lacking systematic reviews in the existing literature. The primary purpose is to lay the groundwork for promoting mental health and preventing procrastination as risky behavior. This systematic review examined five areas related to procrastination and its influence on healthy lifestyle habits in adolescents: technology and procrastination; sleep and procrastination; academic procrastination; and the COVID-19 pandemic. The findings highlight that technology misuse is linked with procrastination; adolescents tend to procrastinate when going to sleep; academic procrastination negatively impacts long-term educational achievements, and the COVID-19 pandemic has exacerbated this phenomenon. Ultimately, it is concluded that procrastination is related to all these aspects and has detrimental effects on adolescents’ physical and psychological development.

## 1. Introduction

When delving into the psychological aspects of procrastination, self-regulation problems become evident [1,2]. The mechanism of standard procrastination follows a specific pattern based on feelings of guilt or discomfort for not performing a particular action, compensation by doing a less relevant task, and justifying not doing the initial task by being engaged in other things (self-deception by doing different tasks) [3].

When delving into the psychological aspects of procrastination, self-regulation problems become evident [1,2]. The mechanism of standard procrastination follows a specific pattern based on feelings of guilt or discomfort for not performing a particular action, compensation by doing a less relevant task, and justifying not doing the initial task by being engaged in other things (self-deception by doing different tasks) [3]. This behavior is not merely about poor time management but is deeply rooted in emotional regulation and cognitive biases [4]. Understanding procrastination requires exploring these underlying psychological mechanisms, which often involve excessive perfectionism, anxiety, and fear of failure [5,6,7].

Procrastination, therefore, is a multifaceted behavior influenced by various emotional and cognitive factors. For instance, perfectionism is characterized by setting unrealistically high standards and critical self-evaluations, often leading to procrastination as individuals avoid tasks they fear they cannot perform perfectly [5]. Anxiety plays a pivotal role, creating procrastination–anxiety–depression axis where the initial delay induces anxiety, which, in turn, reinforces further procrastination and can eventually lead to depression [8]. Adolescents, being in a critical developmental stage, are particularly vulnerable to these patterns, making it essential to understand and address procrastination within this demographic [9,10].

Studies on procrastination focus on the frequency of the habit of procrastinating; it is less common for studies to focus on the reasons why a task is delayed over time [4]. The scientific literature describes multiple causes, but all of them relate to the lack of management of the emotion associated with the delayed task and, generally, to a deficit in self-regulation [4]. Among the most described are procrastination due to excessive perfectionism [5], drowning in negative feelings such as anxiety [6], and avoidance due to fear of failure [7]. The approach, focused on diagnosis, assistance, or prevention, from which the problem is to be addressed, requires deep reflection and investigation of the causes that justify such behavior [4]. In this sense, it is fundamental to address the elements that explain it:

(a) Perfectionism, fear of failure, and procrastination

Perfectionism is defined as “the establishment of excessively high and often unrealistic performance standards, compulsive and incessant efforts to reach these levels, measuring self-esteem largely or entirely in terms of achievement, leading to self-criticism” [5] (p. 972). Several authors relate procrastination and perfectionism [1,5,7]. Some, like [7], link these two terms with negative characteristics such as fear of failure, stress, anxiety, and depression. As ref. [1] define narcissism as a measure of exacerbating procrastination through perfectionism;

(b) Anxiety and procrastination: the procrastination–anxiety–depression axis

According to [6] (p. 91), “Anxiety is the state of agitation, restlessness, and anxiety of the spirit”. These authors define a clear axis that relates procrastination and anxiety; in the first phase of procrastination, postponing a vital task generates a feeling of discomfort, increasing the likelihood of exhibiting symptoms compatible with psychological distress. In turn, this anxiety hinders the resumption of postponed activities. As ref. [8] point out, “People who experience negative emotional states such as depression and anxiety may delay behavior directed at long-term goals” (p. 17).

It is an endless vicious circle: individuals prone to anxious emotional states are more likely to voluntarily postpone [8]. Postponement feeds anxiety (rumination), and anxiety reinforces procrastination. This spiral can lead to a chronic situation, leading to cases of depression [8]. 

### 1.1. Procrastination, a Threat to a Healthy Lifestyle in Adolescence

Adolescence is a stage during which many aspects of physical growth and brain maturation are shaped. Still, it is also a period during which young people tend to adopt unhealthy habits more strongly [9]. During this time, many youths develop behaviors that can impact their short—and long-term health and even influence their adult life [10]. 

The tendency to adopt risky behaviors combines with the tendency to procrastinate. Approximately 20–25% of the general population tends to procrastinate [4,11], and these figures significantly increase in the educational environment, where some studies indicate that up to 70% of students are affected by procrastination [8], while others assert percentages of 80% [4]. It has been observed that 98% of adolescents procrastinate, with 60% at high levels and 10% at very high levels [8].

The effects of procrastination are evident in multiple aspects of life and can directly or indirectly impact mental health, personal well-being, socialization, and social and occupational integration. Today, these consequences are of significant concern in both educational and social spheres. 

Therefore, procrastination and the tendency to adopt risky behaviors become harmful to adopting a healthy lifestyle. This is particularly concerning given the developmental stage of adolescence, where establishing healthy habits is crucial for long-term well-being. Adolescents are at a critical juncture where the foundation for future behavior patterns is laid, making the need for effective interventions to reduce procrastination and promote healthier choices even more pressing.

In our study, we found that the use of technology, sleep patterns, and academic performance are significantly impacted by procrastination. These findings align with previous research indicating that adolescents’ increasing screen time is linked with higher rates of procrastination, poorer sleep quality, and reduced academic achievement. The psychological impact of these behaviors cannot be understated, as increased anxiety and depression are common among those who procrastinate regularly.

Considering the broader societal and educational contexts when addressing procrastination is essential. For instance, the rapid shift to online learning during the COVID-19 pandemic has exacerbated existing issues, highlighting the need for schools to implement supportive measures that encourage time management and self-regulation skills. As researchers, we advocate for the integration of cognitive–behavioral strategies within educational curricula to help students develop these essential skills.

Our findings underscore the importance of early intervention. Programs aimed at improving self-regulation and reducing anxiety could play a pivotal role in mitigating the negative effects of procrastination. Additionally, parental involvement is crucial. Parents must be aware of their children’s procrastination habits and work collaboratively with educators to create environments supporting healthy behaviors.

Adolescents are emerging as a significant risk group in this context, and since adolescence is a crucial stage for habit formation, it is essential to pay attention to this collective from families and educational centers to promote healthy habits and teach appropriate lifestyles.

Research on this topic has focused on various habits related to procrastination that significantly affect the development of a healthy lifestyle. These habits include academic procrastination, bedtime procrastination, procrastination in relation to physical activity, inappropriate use of technologies, and procrastination regarding substance abuse.

Academic procrastination, for example, has become a widespread problem among students, affecting their academic performance and psychological well-being [6,12]. This behavior can have long-term consequences in the lives of young people, limiting their educational and professional opportunities.

Bedtime procrastination is also a problematic habit among adolescents. Despite the importance of sleep for physical and mental development, many young people do not receive enough sleep. The tendency to postpone bedtime, often related to activities such as the use of electronic devices, can negatively affect their long-term health. Furthermore, procrastination is also linked to a lack of physical activity; those who procrastinate tend to show a low commitment to physical activity, which can have a negative impact on their overall well-being [12]. A lack of physical activity can contribute to a range of long-term health issues, including obesity and diseases related to lack of exercise.

The inappropriate use of technology is another significant aspect related to procrastination. Adolescents spend increasing amounts of time online, which can lead to excessive Internet and social media use. This problematic use of technologies has been associated with a greater tendency to procrastinate and with mental health problems, such as anxiety and depression [13,14].

Finally, procrastination is also related to substance abuse; numerous studies have shown that those who procrastinate have a greater predisposition to consume substances such as cannabis and alcohol [15,16,17]. These procrastinating behaviors can increase the risk of dependency and psychological problems associated with substance use, creating a pernicious cycle that negatively affects the health of adolescents and can persist into adult life.

### 1.2. Preventing Procrastinating Behavior

Detecting procrastinating behavior is not straightforward, as it often manifests alongside other more apparent or eye-catching behaviors to an observer. Over the past decade, various tools have been developed to address this issue. However, it is essential to recognize that having data on procrastination and its impact on society is meaningless unless accompanied by actions aimed at its prevention and treatment.

As [18] emphasize, despite its high prevalence and negative consequences for health and well-being, there is still no protocol to respond to those exhibiting this behavior adequately. Prevention and promotion of health are presented as pressing aspects that require concrete proposals to intervene and, even more fundamentally, prevent its appearance.

Ref. [19] highlight the importance of focusing interventions on adolescents with a procrastinating profile on motivation and self-regulation. They propose psychoeducational interventions with realistic approaches that aim to improve motivational aspects. These approaches have proven to be effective in the long term. Additionally, ref. [18] also supports the effectiveness of cognitive–behavioral therapy for therapeutic intervention.

As [20] point out, there is a positive relationship between therapy aimed at treating procrastination and the reduction in this behavior. Similarly, psychoeducational therapy offers good results, especially in the student population compared to the general population.

As previously mentioned, procrastination is highly prevalent and comorbid. In response to this situation, online interventions have emerged [18] that have proven highly effective. The development of effective, easily accessible, and affordable tools represents both a challenge and a crucial opportunity to address the repercussions that arise and affect people’s quality of life.

### 1.3. Turning Points in the Study of Procrastination

As previously mentioned, procrastination is a widespread problem with significant consequences in various areas of life, including mental health, physical health, and the tendency to develop addictive behaviors.

Although the term “procrastination” is old, it was not until a few years ago that its scope and consequences in the academic field were identified. A preliminary bibliographic search reveals that, before 2014, publications related to this concept were anecdotal. However, since that year, the number of documents related to procrastination began to increase exponentially yearly. Between 2014 and 2019, there was an almost constant trend marked by a growing focus on health promotion and prevention in schools. From 2020, the number of publications related to procrastination and education has doubled.

The COVID-19 pandemic brought significant attention to the topic of procrastination due to its radical impact on the educational system, one of the most affected sectors. Uncertainty, confinement, and fear were some of the most impactful factors for society, and the pandemic had a negative effect on people’s well-being, leading to an increase in depression, anxiety, and stress [21]. 

The sudden and unprepared transition to an unprecedented situation put the educational system into crisis. Social distancing posed an extraordinary challenge for teaching, forcing students and teachers to rapidly adapt to distance education [22]. This lack of structure in educational regulation may have contributed to an increase in procrastination [21]. 

Furthermore, indirect factors such as increased smartphone use contributed to a rise in procrastination during confinement. The exponential increase in smartphone use, often described as addictive behavior, made staying continuously updated almost inevitable. Excessive phone use has been linked to an increased risk of procrastination [23], and this abuse was also associated with sleep problems, exacerbating symptoms of anxiety and psychological discomfort [24]. 

Although only two of the selected articles specifically addressed the impact of COVID-19 on procrastination, these findings highlight important trends and underline the significant influence of the pandemic on procrastination behaviors. Therefore, while the pandemic increased focus on procrastination, it is one of many factors contributing to the growing body of research in this area.

### 1.4. Justification for the Systematic Review Study

Based on the evidence presented so far, the need to investigate the prevalence and comorbidities associated with procrastination is supported. Adolescents have emerged as a group particularly prone to developing this behavior, which underscores the urgent importance of implementing training strategies and preventive interventions to avoid the increasingly negative consequences observed in recent years.

The tendency to postpone tasks significantly impacts the daily lives of young people, negatively influencing multiple aspects of their lives. The procrastinating personality extends through various spheres of a person’s life. However, the presented background clearly indicates that one of the most severe repercussions falls on psychological health, which, in turn, is linked to the adoption of unhealthy lifestyles, especially in terms of physical activity.

The comprehensive analysis of existing research on this topic has led to the identification of the main objective of this study, focusing on understanding the relationship between procrastination and the deterioration of habits related to a healthy lifestyle in adolescence. To date, no systematic reviews have addressed this topic in the available literature. The ultimate purpose of this study is to lay the groundwork for promoting mental health and preventing procrastination as risky adolescent behavior.

Objectives

General Objective:

- To understand the relationship between procrastination and the decline in healthy lifestyle habits in adolescence;

Specific Objective:

- To establish the foundations for promoting mental health and preventing procrastination as a risk behavior.

## 2. Materials and Methods

### 2.1. Design

This brief systematic literature review was conducted to achieve the stated objective, applying the updated PRISMA declaration protocol [25] to identify and select documents. This methodology aimed to identify, analyze, and assess studies that demonstrated results related to procrastination and its consequences on developing healthy habits in adolescence.

To study these dimensions, concepts, and terms related to lifestyles and quality of life were chosen, including adolescents’ habits, mental health, anxiety disorders, academic performance, etc. These factors affected or conditioned aspects of adolescents’ quality of life.

### 2.2. Inclusion and Exclusion Criteria 

To carry out the systematic review process, specific and rigorous criteria were established for selecting relevant documents. These criteria ensured that the analysis focused on pertinent and high-quality information, allowing for a comprehensive evaluation of the relationship between procrastination and healthy habits in adolescence. See Table 1.

### 2.3. Description of the Consulted Information Sources and Search Descriptors

For the information gathering, a total of five databases were consulted: Web of Science; Scopus; PsycInfo; ERIC; and Dialnet. These databases index the most critical studies on the topic of this work. The multidisciplinary nature of the analysis approach presented requires consulting databases from the fields of mental health, education, and psychology. The fields covered by each database are as follows:

- Web of Science (WOS): a database for Sciences, Social Sciences, Arts, and Humanities;
- Scopus: a multidisciplinary database;
- PsycInfo: a database for the Behavioral Sciences and Mental Health literature;
- ERIC: a database for Psychology and Education Sciences;
- Dialnet: a multidisciplinary Hispanic database.

The keywords were carefully selected using MeSH terms to ensure a precise and relevant search. The keywords included “procrastination”, “adolescent”, “students”, “lifestyle”, and “health”. These terms were used in both English and Spanish to cover a broader range of studies. The formulated search equation was ((students* OR adolescent*) AND (lifestyle OR health*)) AND procrastination. The roots of the terms were used to maximize the breadth of the search and not limit the results to specific word variations.

### 2.4. Document Selection Process

**Figure 1:**
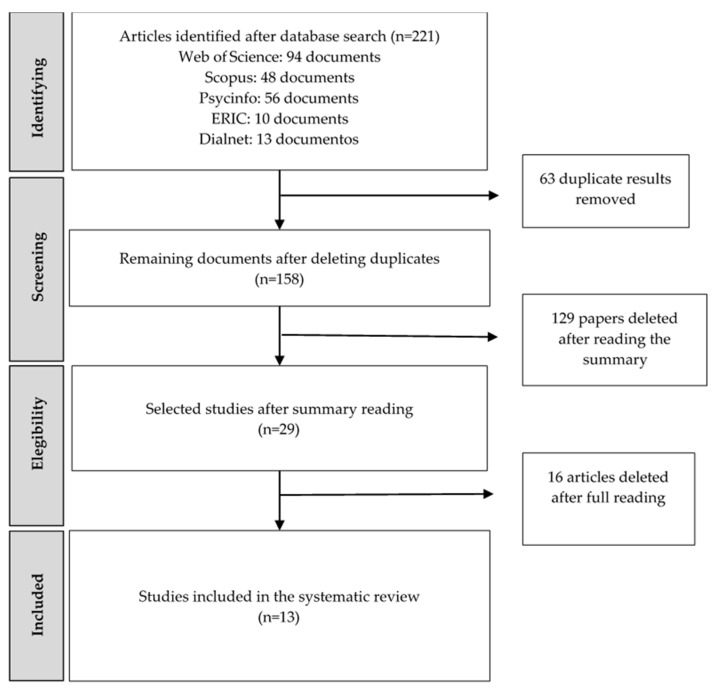
Flow Diagram of the Phases of the Review.

This systematic review commenced in February 2023 and was concluded in July of the same year. After the initial search, the results underwent various filters to obtain the documents that were ultimately included in this review. The process of filtering and selecting articles and studies is described in the flowchart in Figure 1.

The eligibility assessment was carried out independently and in a standardized manner. In the first phase, the search strategy defined in the five databases mentioned was applied, and duplicates were removed using the Mendeley v2.119.0 bibliographic manager. Then, inclusion and exclusion criteria were applied, followed by a review of the documents’ titles and abstracts. Finally, a complete reading of the remaining studies was conducted to verify that they met all the study objectives, resulting in a total of 13 articles and studies.

With the selected documents, a data extraction sheet was developed based on the data extraction template from the Cochrane Consumers and Communication Group [26], as shown in Table 2.

For the analysis and classification of the documents, an analysis was performed to estimate the degree of agreement among the judges, using the Perreault and Leigh agreement coefficient to correct for random effects in this agreement. This coefficient, whose values range from 0 (no agreement) to 1 (perfect agreement), showed a matching value of I = 0.88, indicating a high level of consensus among the judges.

$$S=\left(\frac{Fo}{N}-\frac{1}{K}\right).\left(\frac{K}{K-1}\right)$$

*Fo* = number of judges’ agreements;
*N* = number of elements to be coded;
*K* = total number of categories;

## 3. Results

After selecting the references that met the inclusion and exclusion criteria, the topics linking procrastination with habits that negatively affected a healthy lifestyle were identified. A total of four fundamental areas were identified, from which the results and analysis were presented: (a) technology and procrastination; (b) sleep/rest and procrastination; (c) academic procrastination; and (d) the COVID-19 pandemic and procrastination.

Table 2 synthesizes the relevant data related to this theme. The included documents were published between 2014 and 2023. However, 92% of the included documents were published from 2018 onwards. No articles meeting the criteria were found between 2015 and 2017 inclusive.

The included documents were published in Mexico, Germany, the United States of America, China, the Netherlands, Austria, Lebanon, and the United Kingdom, with 38% of the articles conducted in Germany.

The methodologies used in these studies were varied retrospective, prospective, qualitative, quantitative, etc.

Addressing the identified topics: Technology and procrastination: (a) total of seven articles addressing this theme were included, accounting for 54% of the total; (b) Sleep/rest and procrastination: a total of two documents addressing this theme were included, accounting for 15% of the total; (c) Academic procrastination: one article addressing this theme was included, accounting for 8% of the total; (d) COVID-19 pandemic and procrastination: two articles addressing this theme were included; (e) One article addressed technology, sleep/rest, and procrastination together.

Overall, these studies have revealed that procrastination, whether related to technology, sleep, or academic aspects, negatively impacts adolescents’ academic performance, psychological well-being, and sleep quality. Some studies have highlighted the importance of self-control and willpower in reducing procrastination among the adolescent population. However, it is essential to consider that these results come from diverse studies with different approaches and populations, highlighting the complexity of the phenomenon of procrastination at these ages.

## 4. Discussion

The results indicated that the procrastination levels among adolescents are concerning, with the indices being particularly high in secondary education [27]. Among the causes for this high tendency to procrastinate, the one highlighted by [28] stood out, indicating that adolescents are at the stage of maximum sensation-seeking, thus tending to postpone necessary actions in favor of actions that produce more immediate gratification or a more pleasurable experience. There is a predominance of the preference for “immediate pleasure” over activities or tasks that involve effort or time.

The repercussions of procrastination in adolescents are varied and, in many aspects, parallel to those experienced by adults, as demonstrated by study [30,31]. Additionally, ref. [27] points out that procrastination significantly impacts multiple areas of adolescent development. This phenomenon not only influences physical and psychological health but also manifests in practices that compromise essential aspects such as adequate nutrition, regulated internet use, and physical activity, as indicated by [32,37]. 

Specifically, it has been observed that adolescents who procrastinate tend to experience a deterioration in their psychological functioning. A key finding by [31] is that these young people report elevated levels of stress, which, in turn, negatively impacts their interpersonal relationships, especially within the family environment.

### 4.1. Procrastination and Healthy Lifestyle Habits

In this section, we examine the significant results that highlight the relationship between procrastination and its impact on healthy lifestyle habits. The analysis focuses on several key areas:

- Technology and Procrastination

Currently, adolescents have access to many forms of technology, and its abuse is approached from several perspectives.

Firstly, some studies specifically addressed internet addiction and misuse. Internet addiction is closely related to the phenomenon of procrastination, as indicated by [27]; the greater the internet addiction, the more adolescents exhibit procrastinating behaviors.

Kindt et al. (2019) define Internet Use Disorder (IUD) as a term that encompasses the addictive use of both video games and other Internet applications. The importance of this disorder is such that the American Psychiatric Association includes it as a diagnosis in the fifth edition of the *Diagnostic and Statistical Manual of Mental Disorders* (DSM-5), alongside the diagnosis of “Internet Gaming Disorder” (IGD) [13].

Adolescents suffering from IUDs use the internet as a mechanism to avoid real-life problems and use it to escape from negative mood states. This is especially relevant in the academic sphere, where internet use provides a sense of immediate gratification, while school tasks can be exhausting and even provoke unpleasant feelings, which, in the long term, translates into avoidance and fear of failure. The extent of this problem is immeasurable, as adolescents have constant internet access (through their smartphones and computers), which can be a short-term distraction but can also hinder the achievement of long-term academic goals. “The more students tend to postpone school activities in favor of their online behavior, the harder it will be to start working on them later, as obligations can accumulate and negative feelings associated with them can intensify”. Ref. [13] (p. 11).

However, the consequences of procrastination related to internet addiction did not only affect the educational sphere. As [31] state, multitasking on the internet and insufficiently controlled use in adolescence produce procrastination, which has implications for the psychological functioning of adolescents.

The consequences of the misuse of technologies and procrastination were related by other authors to mental health. As ref. explained that adolescents use social networks as a mechanism to delay tasks and feel that these influence their ability to control distractions, experiencing anxiety and negative moods when they do not have access to their mobile phones. Online relationships become such an important part of adolescents’ social interaction that they describe their peers as antisocial in offline social situations.

As ref. [13] used the concept of nomophobia to refer to the anxiety that adolescents suffer when they cannot contact or access content. As ref [30] used the concept of F.o.M.O (Fear of Missing Out) to define the same fear of missing out. In this case, both authors related it to the use of smartphones. [30] indicated that smartphone addiction in adolescents is mediated by sensation seeking and the fear of missing out, and this leads to procrastination. Additionally, it highlighted that adolescents who procrastinate are more likely to become addicted to smartphones, so there is a direct relationship between FoMO and procrastination.

As refs. [35,38] used group intervention based on cognitive therapy to address IUD and IGD. Ref. [35] used the PROTECT intervention and obtained results that evidenced the reduction in symptoms of both disorders.

On the other hand, refs. [38,39] used the PROTECT+ intervention showed a reduction in symptoms, especially self-reported depression, social anxiety, performance anxiety, and school anxiety, as well as, in general, psychopathology reported by parents;

b. Sleep/Rest and Procrastination

The second area of interest is the relationship between sleep/rest and procrastination. Studies such as those by [28] highlight that procrastination can lead to significant disruptions in sleep patterns among adolescents. Delays in bedtime, often caused by excessive use of technology, result in poorer sleep quality and can negatively affect overall health and cognitive function. 

The adolescents included in their sample delayed going to bed by an average of 45 min. These data worsen in those adolescents with a lack of self-control [28], contrary to adolescents with willpower, who engaged less in bedtime procrastination. However, it was observed that stress affected the quality of sleep, regardless of self-control. Additionally, it was noted that students with self-control did not sleep more because they woke up earlier in the morning. In other words, students with low self-control not only delayed the time to go to bed but also the time to get up.

As described in the background and as indicated by [31], “the quality of sleep is another central aspect for the psychological functioning of adolescents” (p. 7). Therefore, procrastination in this area could potentially be harmful to the development of adolescents. Their experimental study also shows that procrastination is positively related to sleep problems. On the other hand, ref. [33] indicated that sleep deprivation due to a lack of self-regulation interfered with work and decision-making.

Among the triggering factors for bedtime procrastination are stress [28] and the abuse or misuse of technologies [32]. Individuals with low self-control and more impulsive decisions abuse smartphones, increasing the tendency to procrastinate at bedtime [32]. In addition, internet multitasking is one of the behaviors that adolescents adopt to delay bedtime [31];

c. Academic Procrastination

The literature described academic habit as another area in which adolescents procrastinate. Typically, students procrastinate on their studies because they fall into social temptations. That is, the time they should dedicate to tasks is allocated to social activities. This fact causes a negative relationship with the task to be performed (less motivation and enthusiasm for it), which is termed as a “lower positive affect for planned tasks” [29]. This is how procrastination negatively impacts academic performance and is often accompanied by increased stress and anxiety. Adolescents who procrastinate academically are likely to face long-term consequences, including diminished educational and professional opportunities.

The decrease in positive affect toward tasks affected both occasional and habitual procrastinators. However, the latter were more affected by this effect, experiencing more negative than positive affect, as it became difficult for them to find pleasant or meaningful sensations in activities, contrary to social tensions that offered immediate satisfaction. In this respect, short-term self-motivation and mood self-regulation play an important role as they are capable of upregulating positive affect [29];

d. The COVID-19 Pandemic and Procrastination

The COVID-19 pandemic has also played a significant role in shaping procrastination behaviors. The sudden shift to online learning and increased screen time during the pandemic have exacerbated existing procrastination issues, as shown in studies by [21,22]. This period highlighted the urgent need for effective time management and self-regulation strategies in educational settings.

During this period, adolescent students spent more than seven hours in front of screens. This fact led to alterations in a healthy lifestyle: development of depression and anxiety; physical inactivity; less healthy diet; and more procrastination at bedtime [37].

Relating all of the above to procrastination, the autonomy granted to students by distance learning translated in many cases into procrastination due to the great flexibility, lack of routine structure, and the difficulty for teachers in monitoring students and identifying difficulties in self-regulating autonomy [36]. 

The study conducted by [37] demonstrated the following negative consequences of COVID-19 on the lifestyle of adolescent students:

- Female students presented higher depression and anxiety than male students;
- Insomnia, anxiety, and depression were more frequent in those who abused smartphones;
- Spending more than 7 h in front of the screen per day increased depression (67.9%), anxiety (61.6%), insomnia (82.1%), and procrastination at bedtime;
- It also indicated a shift toward a less healthy diet and light exercise.

As indicated by [37], although this study analyzed the problem from the 7 h of screen use, “these findings should be taken into account when defining appropriate screen time and helping students to control the time they spend in front of the screen” (p. 107).

### 4.2. Consequences of Procrastination on Healthy Lifestyle Habits

The levels of procrastination in adolescents are alarmingly high, which has negative consequences in all spheres of development, affecting physical health, psychological well-being, and healthy lifestyle habits. This reinforces the urgent need for targeted interventions and comprehensive strategies to address this pervasive issue.

The available studies for this age group focus particularly on technologies [13,27,30,31,32,34,35,38]. They argue that uncontrolled use of technology in all its forms (internet, smartphones, etc.) is associated with procrastination, affects healthy lifestyle habits, and can have repercussions on the mental health of adolescents. The easy and continuous access to these resources and the ease of obtaining gratification from them cause adolescents to postpone less rewarding tasks [13]. 

Sleep and rest are other healthy lifestyle habits that adolescents postpone [28,32,33]). The lack of sufficient rest has negative repercussions on adolescents’ physical and psychological development [31]. In many cases, bedtime is delayed for more satisfying activities related to the uncontrolled use of technologies [32]. 

The negative repercussions of procrastination do not leave the academic sphere indifferent. The search for pleasant sensations and the lack of self-control interfere with educational development and can have long-term consequences. Again, the abuse or misuse of technologies interferes with this aspect [29]. 

Therefore, it has been demonstrated that the abuse/uncontrolled use of technologies was associated with procrastination and had negative repercussions on healthy lifestyle habits, both directly and indirectly, even leading to negative consequences for the mental health of adolescents.

It is possible that this is why the interventions found focus on addressing disorders resulting from internet use [35,38].

### 4.3. Limitations of the Systematic Review of the Literature

This systematic review presents a series of limitations. Firstly, although there is an abundant literature addressing the procrastination of healthy life habits in early childhood, similar research focusing specifically on adolescence is scarce. Consequently, no articles meeting the criteria that specifically address physical activity, healthy eating, or substance abuse were found for adolescents. This gap in the literature underscores the need for more targeted research in this age group. Furthermore, the analysis revealed several methodological limitations. Many reviewed studies have limited sample sizes and lack diversity, making it difficult to generalize the results to a broader adolescent population. Additionally, the research design of many studies is cross-sectional or retrospective, which hinders the establishment of causal relationships. Longitudinal studies that follow adolescents over time would provide a better understanding of the evolution of procrastination.

Furthermore, this analysis revealed several methodological limitations. Many reviewed studies have limited sample sizes and lack diversity, making it difficult to generalize the results to a broader adolescent population. Additionally, the research design of many studies is cross-sectional or retrospective, which hinders the establishment of causal relationships. Longitudinal studies that follow adolescents over time would provide a better understanding of the evolution of procrastination.

Another significant limitation is the reliance on self-report questionnaires to collect data on procrastination. These instruments are subject to response bias and may not always accurately reflect adolescents’ actual behavior. Moreover, many studies do not adequately control for multiple variables that can influence procrastination, such as socioeconomic, cultural, or gender factors, which can confound the results.

There is also a lack of intercultural studies, as most research focuses on specific cultural contexts like North America or Europe. Intercultural research would be valuable in understanding how procrastination varies across different cultures. Additionally, despite the high incidence of procrastination and its potential negative consequences, there is a scarcity of studies and tools that focus on the prevention and treatment of procrastination in adolescents. Most studies identify procrastination and its associated factors but often do not offer practical solutions or concrete interventions to address this behavior. Lastly, while aspects such as technology use, sleep, and academic procrastination have been investigated, other important factors may not have been explored in detail. Future research should aim to address these unexplored factors to provide a more comprehensive understanding of procrastination in adolescents.

In summary, although the reviewed studies provide valuable information about procrastination in adolescents, it is important to consider these limitations when interpreting the results and recognize that more research is needed to fully understand this phenomenon and develop effective prevention and treatment strategies.

## 5. Conclusions

The present work reveals alarming data about procrastination among adolescents, especially those attending Secondary Education. Several healthy life habits and areas in which adolescents tend to procrastinate have been identified, and it has been concluded that procrastination is closely related to the abuse and misuse of technology, whether in the form of internet addiction or excessive smartphone use. The negative consequences of procrastination extend to all areas of adolescent development, affecting the ability to maintain a proper sleep schedule, adhere to a healthy diet, and maintain optimal levels of physical activity. Furthermore, a connection has been observed between procrastination and mental health problems, including insomnia, anxiety, and, in extreme cases, depression.

It is important to note that the consequences of procrastination are not limited to adolescence but can have a lasting impact on young people’s life habits, which, in turn, can lead to health problems in adulthood. However, most reviewed studies did not directly address the long-term implications of these procrastinating habits in adult life nor explored life habits related to global health in adulthood, such as diet and physical activity. This systematic review of the literature supports the connection between procrastination and the decline in healthy life habits in adolescence. However, there is a need for further research to explore the extent of these habits at other life stages and analyze the potential long-term consequences.

Additionally, the lack of studies and tools focused on the prevention and treatment of procrastination is emphasized, highlighting the importance of future efforts in the detection, prevention, and management of these procrastinating behaviors, with a focus on health promotion.

This systematic review is valuable for researchers in related fields as it synthesizes current knowledge, identifies gaps in the literature, and proposes directions for future research. The main contributions of this review are threefold. Firstly, it provides a comprehensive overview of the relationship between procrastination and various aspects of adolescents’ health and lifestyle, offering a holistic understanding that can inform both clinical practice and educational strategies. Secondly, identifying methodological limitations and gaps in the existing research sets the stage for more robust and focused future studies. Finally, this review emphasizes the urgent need for targeted interventions and preventive measures, underscoring the practical implications of the findings for policymakers, educators, and mental health professionals.

## Figures and Tables

**Table 1:** Inclusion and exclusión criteria.

Inclusion Criteria	Exclusion Criteria
Articles addressing procrastination and healthy habits in adolescence	Documents related to healthy habits and adolescence not associated with procrastination
Documents in English and Spanish	Reviews, books, book chapters, or doctoral theses
Documents published from 2014 to the present	Documents published before 2014
Peer-reviewed scientific articles	Articles in languages other than English and Spanish

**Table 2:** Data Extraction Table.

Author/Year	Country	Design	Sample	Topic	Results
Ref. [27]	Mexico	Descriptive–correlative study, quantitative, cross-sectional, nonexperimental	*n* = 702 high school and upper middle school students aged 13–18.	Technology and procrastination	- Participants report above-average procrastination-related behaviors; - Procrastination impacts academics and all areas of development. High school teens procrastinate more than teens with higher levels of study; - The more significant the addiction to the internet, the more adolescents exhibit delaying behaviors.
Ref. [28]	Germany	Retrospective analysis through a daily questionnaire.	*n* = 185 participants (adolescent students)	Sleep/rest and procrastination	- Students included those who missed their ideal bedtime by 45 min; - Negative association between self-control of traits and procrastination at bedtime. Students whose willpower is not limited went to bed earlier and were less involved in procrastination at bedtime; - Sleep quality was negatively affected by stress regardless of the level of willpower.
Ref. [29]	EEUU	Two longitudinal studies, prospective.	*n* = 154 adolescent students	Academic procrastination	- Procrastinating time is related to the more significant existence of social temptations; - Procrastinators report a lower level of positive affection.
Ref. [30]	China	Convenience sampling	*n* = 794 adolescent students	Technology and procrastination	- The search for sensations could positively predict smartphone addiction in teenagers, and this is related to the F.o.M.O and procrastination. Teens are at the maximum stage of seeking sensations
Ref. [31]	Germany	Experimental study, cross-sectional	*n* = 818 adolescent students	Technology and procrastination	- Procrastination was significantly associated with impaired psychological functioning of adolescents in multiple domains and sleep problems. It is associated with multitasking on the internet; - These report low levels of satisfaction in their relationship with their parents; - Adolescents suffer negative consequences of procrastination similar to those of emerging adults or the general population.
Ref. [32]	China	Experimental study, cross-sectional	*n* = 4162 adolescent students	Technology, sleep/rest, and procrastination	- Procrastination at bedtime was related to the future time perspective, dual self-control, and problematic use of smartphones.
Ref. [33]	Netherlands	Prospective study	*n* = 2637 participants from 16 to 93 years.	Sleep/rest and procrastination	- A significant proportion of the general population sleeps less than they would like; - Sleep-deprived individuals achieve worse results in self-regulation tasks involving working memory or decision-making.
Ref. [34]	United Kingdom	Qualitative study	*n* = 42 adolescent students	Technology and procrastination	- Teens experience feelings of procrastination, using social media to delay tasks, and are considered influenced (at least partially) by their ability to control their distraction; - They experienced anxiety and negative moods when they did not have their mobile devices, not being able to contact or access content (nomophobia); - Teens described their peers as antisocial in offline social situations.
Ref. [13]	Germany	Controlled, randomized, longitudinal study	*n* = 418 adolescent students	Technology and procrastination	- Procrastination is closely related to some central clinical symptoms of internet use disorder (IUD); - Students often have constant access to online activities, so rewarding experiences are permanently available. This may have consequences for long-term learning objectives.
Ref. [35]	Germany	Randomized clinical trials by groups	*n* = 422 adolescent students.	Technology and procrastination	- PROTECT is an intervention group based on cognitive behavioral therapy. It targets changes in the processing of addictive rewards and pathological cognitive mechanisms; - PROTECT effectively reduced the symptoms of gambling disorder and unspecified internet use disorder for 12 months.
Ref. [36]	Austria	Retrospective study	*n* = 25,305 adolescent students	COVID-19 pandemic and procrastination	- During the pandemic caused by COVID-19, the autonomy given to students resulted in many cases of procrastination.
Ref. [37]	Lebanon	Cross-sectional study	*n* = 510 adolescent students	COVID-19 pandemic and procrastination	- Insomnia, depression, and anxiety were higher among telephone users; - Among schoolchildren who spent seven hours in front of a screen, they developed depression and anxiety, exercised less, followed a less healthy diet, and procrastinated at bedtime.
Ref. [38]	Germany	Randomized controlled trial	*n* = 54 adolescent students	Technology and procrastination	- The PROTECT+ program is a group intervention program based on TCC for adolescents. Patients showed a significant reduction in the severity of IUD symptoms at 12 months of follow-up.

## Data Availability

The data can be requested from the corresponding.
